# PEALut in the Dietary Management of Patients with Acute Ischemic Stroke: A Prospective Randomized Controlled Clinical Trial

**DOI:** 10.3390/jcm13020509

**Published:** 2024-01-16

**Authors:** Massimo Bonzanino, Marianna Riolo, Iacopo Battaglini, Marilisa Perna, Marco De Mattei

**Affiliations:** 1 S. S. Stoke Unit, Dipartimento Area Medica, Ospedale Santa Croce di Moncalieri, ASLTo5, 10024 Moncalieri, Turin, Italy; 2 S. C. Neurologia, Dipartimento Area Medica, Ospedale Santa Croce di Moncalieri, ASLTo5, 10024 Moncalieri, Turin, Italy

**Keywords:** acute ischemic stroke, ultramicronized Palmitoylethanolamide, luteolin, neurological deficit, disability, cognitive impairment

## Abstract

Acute ischemic stroke (AIS), which represents 87% of all strokes, is caused by reduced blood supply to the brain associated with a prolonged inflammatory process that exacerbates brain damage. The composite containing co-ultramicronized Palmitoylethanolamide and luteolin (PEALut) is known to promote the resolution of neuroinflammation, being a promising nutritional approach to contrast inflammatory processes occurring in AIS. This study included 60 patients affected by acute ischemic stroke and undergoing thrombolysis. PEALut 770 mg was administered to 30 patients, twice daily for 90 days, in addition to the standard therapy. Neurological deficit, independence in activities of daily living, disability and cognitive impairment were investigated. In all patients, the severity of AIS defined by the NIHSS score evolved from moderate to minor (*p* < 0.0001). Patients’ independence in daily living activities and disability evaluated using BI and mRS showed a significant improvement over time, with a statistically significant difference in favor of PEALut-treated patients (*p* < 0.002 for BI, *p* < 0.0001 for mRS), who achieved also a marked improvement of cognitive function evaluated using MMSE and MoCA tests. PEALut proved to be a safe and effective treatment in addition to thrombolysis in the management of patients with acute ischemic stroke.

## 1. Introduction

The occurrence of stroke, the second leading cause of death and the leading cause of disability worldwide, is on the increase with one in four people experiencing stroke in their lifetime [1]. The impact of stroke on people’s lives represents an important challenge for society: in addition to being a sudden event, stroke affects the individual who is unprepared to deal with the process of rehabilitation or the disabilities resulting from this condition [2]. Acute ischemic stroke (AIS), in particular, accounts for 87% of the total incidence of stroke and is characterized by a sudden cessation of oxygen and blood supply due to arterial occlusion in local cerebral tissue [3]. The transient or permanent occlusion of cerebral vessels leads to brain infarcts, along with cerebral tissue death and focal neuronal damage [4]. The brain responds to the ischemic injury with an acute and prolonged inflammatory process, characterized by the rapid activation of microglia and astrocytes, production of proinflammatory mediators and infiltration of various types of inflammatory cells (including neutrophils, different subtypes of T cells, monocyte/macrophages) into the ischemic region, exacerbating brain damage [5,6,7,8]. Currently, to treat patients after ischemic stroke, reperfusion therapy with thrombolytic drugs such as intravenous tissue plasminogen activator or mechanical thrombectomy is used. However, these therapies have many limitations, and the rehabilitation of patients is frequently insufficiently effective and in many cases does not restore lost functions [3,9]. Since neuroinflammation plays a significant role in the evolution of the ischemic event, its modulation may help to reduce neuronal injuries and attenuate cerebral ischemic damage. Mast cells and glia are endowed with endogenous homeostatic mechanisms/molecules that are involved in the mechanisms activated in the body as a result of different types of tissue damage [10]. The endogenous lipid amide Palmitoylethanolamide (PEA), which belongs to the family of N-acylethanolamines, has been found in mammalian tissues, especially the brain, and is released “on demand” during injury to counteract neuronal damage [11,12]. Thus, PEA accumulates in tissues and exerts a local, autacoid, anti-injury function via modulating mast cells and microglia, protecting neurons against excitotoxicity, reducing tissue inflammation, decreasing hyperalgesia and exerting a neuroprotective function [13,14]. The release of endogenous PEA, observed in the penumbral tissue surrounding the primary ischemic lesion within the first day after stroke, was reported in a case-report study of a patient with hemispheric stroke [15]. Moreover, it was demonstrated that PEA achieved a significant neuroprotective effect by reducing the size of infarcted tissue after transient middle cerebral artery occlusion (tMCAO) in an animal model of acute stroke [16] and significantly reducing the number of cells undergoing apoptosis and inflammation in the brain in an ischemia-reperfusion rat model [17].

When given orally, PEA presents limitations in terms of solubility and bioavailability, so it is micronized (mPEA) and/or ultramicronized (umPEA) to improve its biological efficacy [18,19]. Furthermore, it has been demonstrated that the association between PEA and some natural molecules like luteolin or polydatin through co-(ultra)micronization processes [20] produced synergistic effects, improving the neuroprotective effect of umPEA by acting simultaneously on the phenomena of inflammation and the formation of reactive oxygen species [14,21]. Importantly, the first evidence of the administration of the co-ultramicronized composite of PEA with luteolin (co-ultraPEALut, or just PEALut) in the post-acute stroke phase was obtained in a cohort of 250 patients undergoing neurorehabilitation. The 60-day nutritional supplementation with PEALut improved the neurological status, cognitive abilities, the degree of spasticity, pain and independence in daily living activities with an excellent tolerability profile already after 30 days of treatment [22]. Based on this evidence, with this prospective, randomized, controlled clinical trial, we wanted to investigate whether the nutritional supplementation with the neuroprotective composite PEALut, in addition to thrombolytic therapy, could provide a better functional and cognitive outcome compared to thrombolysis alone, when administered in the acute phase of ischemic stroke.

## 2. Materials and Methods

### 2.1. Study Participants

The study involved a total of 60 patients affected by AIS and undergoing thrombolytic therapy at the Stroke Unit of Santa Croce Hospital in Moncalieri (Turin, Italy). Patients of both sexes who experienced AIS and underwent thrombolysis were eligible for the study if they were ≥18 and ≤90 years old and had a National Institutes of Health Stroke Scale (NIHSS) score between 5 and 20 and no significant pre-stroke disabilities (they were able to carry out all usual duties and activities before the ischemic event). Patients with hemorrhagic stroke or concomitant diseases which could interfere with these evaluations, were excluded from the study.

This study was approved by the Ethics Committee “A.O.U. San Luigi Gonzaga” of Orbassano (Turin, Italy) (prot. No. 7738 of 28 May 2020). Informed consent was obtained from all patients. The study was conducted in agreement with the Declaration of Helsinki about ethical principles for medical research involving human subjects and according to the Good Clinical Practice (GCP).

### 2.2. Study Design

This study was a prospective, randomized, open-label, controlled, clinical trial. Eligible patients were randomized into two groups in a 1:1 ratio using a computer-generated randomization list. Out of a total of 60 patients, 30 were treated with thrombolytic therapy alone (alteplase), and 30 received in addition supplementation with the Food for Special Medical Purposes (FSMP) PEALut (Glialia^®^, Epitech Group SpA, Saccolongo, Padova, Italy), in its oral suspension formulation (PEA 700 mg + luteolin 70 mg in 10 mL) 20 mL/day (10 mL twice daily), starting within 72 h after ischemic event onset. PEALut dosage was prescribed according to its indications for use and the existing literature data [22,23]. For those patients unable to take oral formulations, PEALut supplementation was administered via nasogastric tube. All patients were admitted to the Stroke Unit and were subjected to the standard medical practice both for the etiopathogenesis of stroke and for the pharmacological (anticoagulant/antiplatelet agent/antihypertensive drugs, where indicated) and rehabilitation therapy.

### 2.3. Outcome Measures

All patients underwent the following evaluations at baseline (T0), at discharge from the Stroke Unit (on average after 10 days, T1) and after 90 days (T2), by the same physician who was comprehensively trained for the administration of the validated tools and the data collection:

(i) Neurological deficit was evaluated at T0, T1 and T2 using the 11-item NIHSS, whose scores ranged from 0 to 42, with higher scores indicating more severe neurological deficit [24,25];
(ii) Independence in activities of daily living and degree of disability were evaluated at T0, T1 and T2 using Barthel Index (BI) [26] and modified Rankin Scale (mRS), respectively [27]. The BI maximal score is 100, indicating that the patient is fully independent in physical functioning, while the mRS scale consists of 6 grades from 0 to 5, with 0 corresponding to no symptoms and 5 corresponding to severe disability [28];
(iii) Cognitive impairment at T1 and T2 was evaluated using Mini-Mental State Examination (MMSE) [29] and Montreal Cognitive Assessment (MoCA) [30]. For MMSE, a perfect score is 30 points, a score of 24 is the recommended score, and a score of 23 or lower indicates dementia. Like the MMSE, the MoCA is a brief 30-point assessment with a proposed cut-off score of 26 and a ≤ 25 score indicative of cognitive impairment [31].

### 2.4. Safety Assessments

At T0 and T2, patients were subjected to routine blood chemistry and hematology analyses and monitored for possible occurrence of adverse events throughout the course of the study.

### 2.5. Sample Size Calculation and Statistical Analysis

Assuming a 2-point difference on the NIHSS scale between the control and PEALut group, the power analysis (beta = 0.20 and alfa = 0.05) revealed that a sample of 27 subjects per group was needed, which was rounded to 30 to handle possible dropout.

Data analysis was conducted using the Generalized Linear Mixed Model (GLMM). Variables such as gender and age were included in the model as covariates. The distribution of patients, regarding the days spent in the Stroke Unit and the comorbidities, was performed using the Fisher or Kruskal–Wallis test. The comparison between the groups at the different time points was assessed by a post hoc analysis performed using the Tukey–Kramer multiple comparisons test. Values are expressed as mean ± standard error (S.E.) or standard deviation (S.D.), as specified. A *p*-value of less than 0.05 was considered statistically significant.

## 3. Results

### 3.1. Patients Baseline Characteristics

A total of 60 patients (27 females and 33 males) with a mean age ± S.D. of 77.9 ± 10.30 years, admitted to the Stroke Unit of Santa Croce Hospital in Moncalieri between September 2020 and April 2022, were enrolled in this study and randomized to receive PEALut in addition to thrombolysis or thrombolytic therapy alone. All PEALut patients completed the study period with a treatment adherence of 100%, while in the control group, one patient died between T1 and T2 due to the worsening of its clinical conditions, and one did not show up for the T2 follow-up visit and was considered lost to follow-up.

Control and PEALut groups were homogeneous for the number of days spent in the Stroke Unit (T0 -T1 period), stroke site and comorbidities distribution (with the only exception of non-ischemic heart disease). The majority of patients suffered from anterior circulation stroke (93% in control and 90% in PEALut-treated group), and seven patients in each group (23%) suffered from previous transient ischemic attack (TIA). Among all patients, the most prevalent comorbidity was hypertension found in 70% of controls and 87% of PEALut patients, whereas a medical history for heart failure, neoplastic disease, ischemic heart disease and diabetes was seen in a total of 7, 10, 12 and 14 patients, respectively.

Control and PEALut groups were also homogeneous at baseline for the severity of AIS (NIHSS score), independence in daily living activities (BI score) and disability (mRS score).

Detailed patients baseline characteristics are reported in Table 1.

### 3.2. National Institutes of Health Stroke Scale (NIHSS)

**Figure 1:**
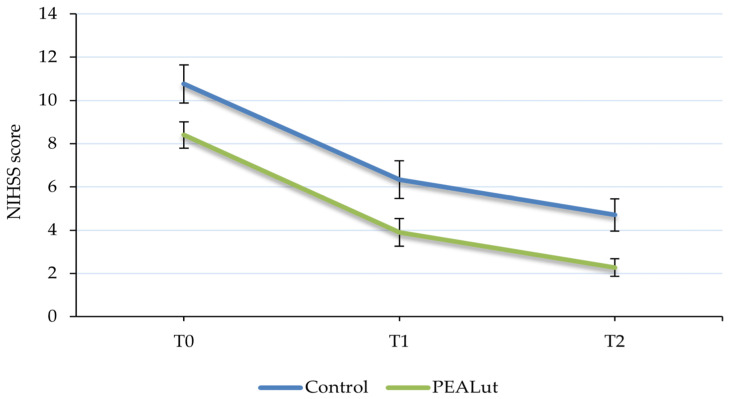
Evolution of National Institutes of Health Stroke Scale (NIHSS) score over time. The severity of stroke improved over time, evolving from moderate to minor, in all patients with no significant difference between the two groups. Values are presented as mean ± S.E.

Changes in NIHSS scores were evaluated at each time point showing a significant improvement over time in both groups (*p* < 0.0001). Among the control patients, stroke deficit severity improved progressively throughout the observation period: the mean NIHSS score decreased from 10.8 ± 0.88 at T0 to 6.3 ± 0.87 at T1 and 4.7 ± 0.74 at T2. Similar improvements occurred in patients who received PEALut, with a mean NIHSS score of 8.4 ± 0.61 at baseline, 3.9 ± 0.64 at T1 and 2.3 ± 0.41 at T2 (Table 2 and Figure 1).

### 3.3. Barthel Index (BI)

**Figure 2:**
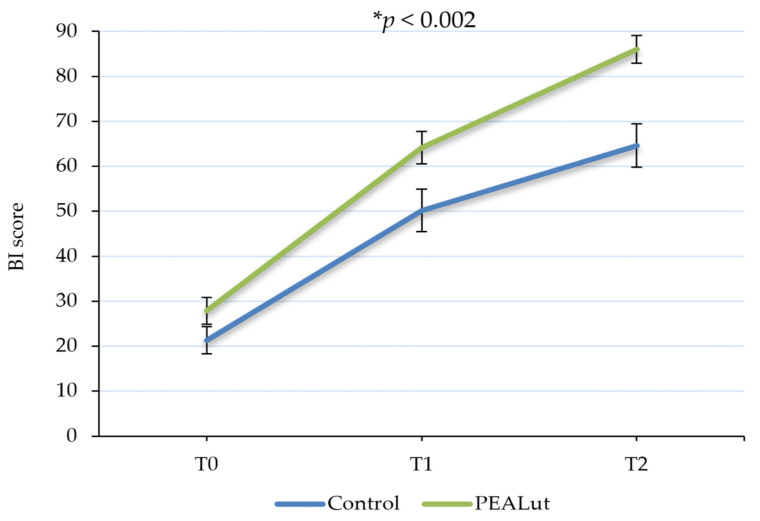
Evolution of Barthel Index (BI) score over time. Patients’ independence in daily living activities significantly improved over time. A statistically significant difference between the two groups was observed in favor of PEALut-treated patients. Values are presented as mean ± S.E. * Significant change over time (GLMM).

Patient independence and mobility in daily living activities evaluated using BI showed a significant improvement over time, with a statistically significant difference between the two groups in favor of the PEALut-treated one (*p* < 0.002). In patients who received only thrombolysis, the mean BI scores were 21.3 ± 3.06 at T0, 50.2 ± 4.72 at T1 and 64.6 ± 4.81 at T2, whereas in patients who received thrombolysis + PEALut, the mean scores were 27.8 ± 2.96, 64.2 ± 3.62 and 86.0 ± 3.06 at T0, T1 and T2, respectively. Furthermore, the post hoc analysis highlighted that the BI score improvement of patients who received PEALut was greater in comparison to that obtained by control patients, almost reaching a statistical significance between the two groups (*p* = 0.0539) at the end of the observation period (Table 3 and Figure 2).

Among the considered covariates, age showed a significant influence (*p* = 0.0001) on the results, with older patients achieving less noticeable results in the improvement of their independence.

### 3.4. Modified Rankin Scale (mRS)

**Figure 3:**
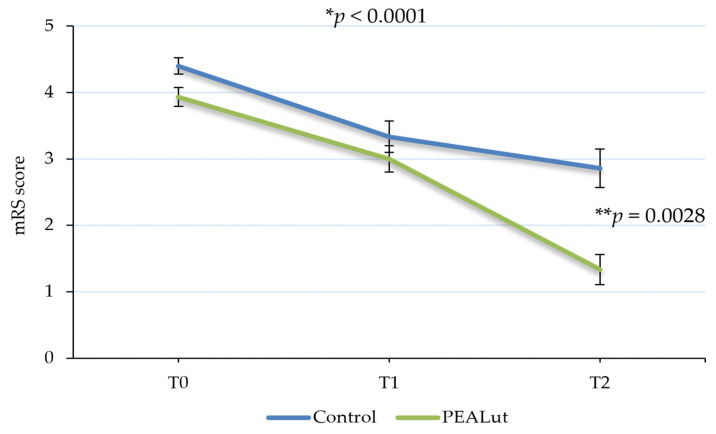
Evolution of modified Rankin Scale (mRS) score over time. Patients’ disability significantly improved over time, with a statistically significant difference between the two groups in favor of PEALut-treated patients. Values are presented as mean ± S.E. * Significant change over time (GLMM); ** significant change between the two groups (post hoc analysis).

The functional independence detected using mRS showed a significant improvement over time, with a statistically significant difference between the two groups in favor of the PEALut-treated one (*p* < 0.0001). In the control group, the mean mRS total score decreased from 4.4 ± 0.12 at T0 to 3.3 ± 0.24 at T1 and 2.9 ± 0.29 at T2. In the PEALut-treated group, the average value of mRS at baseline was 3.9 ± 0.14, which decreased to a mean value of 3.0 ± 0.20 at T1 and a mean score of 1.3 ± 0.23 at T2. Importantly, post hoc analysis showed that at the end of the treatment, the mean BI scores of the two groups reached a statistically significant difference (*p* = 0.0028) (Table 4 and Figure 3).

Moreover, the functional independence was influenced by age: younger patients obtained in fact better results (*p* = 0.0027).

### 3.5. Mini-Mental State Examination (MMSE) and Montreal Cognitive Assessment (MoCA)

At T1, patients considered for cognitive examination were 30 in the control group and 29 in the PEALut one (1 patient was illiterate), whereas at T2, patients were 26 in the control group (2 patients did not show up for examination, 1 patient died due to the worsening of its clinical conditions, 1 patient was unable to carry out the tests due to the worsening of its oncological condition) and 28 in the PEALut-treated group (1 illiterate and 1 patient unable to carry out examinations for hospitalization in another structure) (Table 5).

At T1, after an average of about 10 days from hospitalization, only 9 patients (30%) of the control group were able to perform the MMSE test, while 20 patients (69%) of the PEALut group completed the assessment. At the end of the observation period (T2), patients able to perform MMSE dropped to 5 in the control group (19.2%) and increased to 21 (75%) in the PEALut group (Table 2).

Similar results were obtained for MoCA examination, where only 8 patients (26.7%) were able to perform the test at T1 in the control group, against 19 in the PEALut group (65.5%). At T2, patients decreased to 3 in the control group (11.5%) and remained almost unchanged in the PEALut-treated group (64.3%) (Table 5).

In the PEALut-treated group, the MMSE mean score improved from 23.7 at T1 to 25.5 at T2, while the MoCA mean score changed from 19.1 at T1 to 23.3 at T2. No analysis of MMSE and MoCA scores was made in the control group and between the control and treated groups due to the small number of subjects who performed cognitive tests at T2. The MMSE score was not corrected for age and scholarity due to the lack of data about patients’ educational level.

### 3.6. Treatment Safety and Tolerability

No PEALut-related adverse events were observed over the course of the study. One patient in the control group died between T1 and T2 due to the worsening of its clinical condition. Routine blood chemistry and hematology analysis did not reveal any deviations from their normal ranges in relation to PEALut treatment.

## 4. Discussion

AIS affects millions of individuals worldwide, with a significant impact on mortality and disability rates. According to the World Health Organization, it is a leading cause of death and disability globally, making effective interventions crucial to reducing its impact.

The major contributors to ischemic neuronal injury are oxidative stress and inflammation, with a critical involvement of immune-system-mediated neuroinflammation in determining the fate of the brain following ischemic stroke [32]. In consideration of these findings, this study investigated whether supplementation with the anti-neuroinflammatory compound PEALut (FSMP, Glialia^®^, Epitech Group SpA), starting from the first hours after the ischemic event, could improve the neurological and functional status of stroke patients undergoing thrombolysis.

Stroke deficit severity is highly labile in the first few hours of onset. Substantial spontaneous improvements during the first 24 h occur in nearly 40% of patients and are twice as common as spontaneous worsening [33]. However, the functional recovery of stroke patients is associated with the severity of initial neurological damage which can be quantitatively measured and classified using the NIHSS scale [34]. Moderate and severe stroke types exhibit a low degree and slow rate of functional recovery compared with mild stroke [35]. To understand the level of neurological deficit in control and PEALut groups, the NIHSS scores at each time point were examined. At baseline, all the patients presented a stroke of moderate severity, which at the end of treatment evolved into minor stroke, indicating a recovery in neurological deficit in both groups over time.

A major focus in stroke recovery studies is functional independence, as 26% of ischemic stroke patients remain disabled in basic activities of daily living (ADL), and 50% experience declined mobility due to hemiparesis [36]. In this study, BI and mRS scores were used as ADL assessment scales to understand the level of functional recovery in both groups at each time point. The independence in activities of daily living improved in all patients over time, with a significant increase in the PEALut group compared to controls. Patients treated with PEALut showed a recovery of daily autonomy evident already at the first time point, after about only 10 days after the ischemic event. It should be noted that in the control group, the mean BI score was 21.3 at T0, reaching a maximum average score of 64.6 at T2, whereas with PEALut treatment, a mean BI score of 64.2 was reached already at T1, indicating an earlier and faster recovery. A further improvement was registered at T2, where BI reached a mean score of 86 out of 100 points in the PEALut-treated group. The independence in daily activities results is consistent with the degree of disability: at T0, both groups had, approximately, an mRS mean score of 4, corresponding to a patient with moderately severe disability, unable to attend to own bodily needs without assistance or unable to walk unassisted. In the control group, the mRS score at T1 was reduced to a mean value of 3.3 and to 2.9 at T2, which describes a still-present moderate disability. On the other hand, the result obtained with PEALut at the second follow-up is relevant, when mRS reached a score equal to 1.3, indicative of no significant disability, with patients able to carry out all their usual activities. The significant improvement of both BI and mRS scores compared to the treatment carried out with thrombolysis alone confirmed the neuroprotective and anti-neuroinflammatory properties of PEALut and its role in the improvement of neurological conditions.

The inflammatory cascade creates an unfavorable environment for tissue recovery; thus, if the tissues could be “cleaned from inflammation”, a favorable environment could be created for their regeneration [37]. This was demonstrated in animal models of neurodegeneration, in which umPEA improved neurobehavioral functions, including memory and learning, by reducing oxidative stress and pro-inflammatory and astrocyte marker expression and promoted neurogenesis, neuronal viability and survival, inhibiting mast cell infiltration/degranulation and astrocyte activation [38,39]. Increasing available clinical data suggest that dietary management with m/umPEA improves global executive function, working memory, language deficits and daily living activities and reduces cognitive impairment [38,39]. In particular, the neuroprotective potential of PEA was reported by Ahmad et al. in an animal model of acute stroke: PEA reduced infarct size, blocked the infiltration and activation of astrocytes, reduced apoptotic cell death and pro-inflammatory marker expression and improved neurobehavioral functions, as determined by monitoring motor deficits [40]. In addition to this, PEALut association has been proved to significantly decrease the neuroinflammatory and apoptotic pathways, modulate the activation of astrocytes and microglia, decrease the oxidative stress and promote neuronal regeneration in different experimental models of vascular dementia, brain and spinal cord injury [41,42,43,44]. Another more recent translational evidence reported that PEALut was able to produce neuroprotective effects in a transient middle cerebral artery occlusion animal model and improve the neurological status, degree of spasticity, cognitive abilities, pain and independence in daily living activities in a cohort of 250 stroke patients, after only 30 days of treatment [22].

Further, among stroke survivors, cognitive impairment is present in more than 40% of patients [45], and cognitive improvements mostly occur in the first 3 months after stroke. MMSE and MoCA tests are both considered good screening tools for cognitive impairment at 3 months post-stroke [46]. Our results suggest a beneficial effect of PEALut in ameliorating cognitive impairment, as patients treated with this supplement were able to perform both MMSE and MoCA tests, compared to patients treated with thrombolysis alone. After an average of 10 days from hospitalization, 69% of patients in the PEALut-treated group were able to perform MMSE examination versus only 30% of the controls, while at the end of the observation period, patients dropped to 19.2% in the control group and increased to 75% in the PEALut one. Similar results were obtained from MoCA examination, where 26.7% of patients were able to perform the test at T1 in the control group, decreasing to 11.5% at T2, against 65.5% at T1 and 64.3% at T2 in the PEALut-treated group. In PEALut patients, the MMSE mean score improved from a mean value of 23.7 at T1 to 25.5 at T2, whereas MoCA score improvement was 4.2 points, which exceeds the data reported in the literature and the MCID (Minimal Clinically Important Difference, approximately 2 points) for stroke rehabilitation patients [47,48,49].

The role of umPEA in the context of cognitive decline was investigated particularly in neurocognitive disorders, highlighting an improvement of cognitive symptoms following umPEA administration. In an animal model of Alzheimer’s disease (AD), the chronic (three months) umPEA administration rescued cognitive deficit and restrained neuroinflammation and oxidative stress [50]. Moreover, umPEA induced considerable improvements in cognitive and neural function also during both the early pre-symptomatic and later-symptomatic stages of AD, restraining the cognitive/behavioral decline by exerting a combination of anti-neuroinflammatory and neuroprotective effects [51]. As for human studies, a case report evaluated PEALut effects on mild cognitive impairment (MCI) symptomatology, reporting progressive symptom amelioration during the 9-month therapy, with a significant improvement of neuropsychological performances at the end of treatment [52]. Similarly, in another study, patients with MCI receiving PEALut showed a significant improvement in short-term memory, suggesting a slowdown of cognitive symptoms and an improvement in non-cognitive behavioral disturbances, which are associated with a higher risk of dementia [53]. Significant amelioration in the behavioral symptoms has been also observed after a 4-week treatment with PEALut in patients affected by frontotemporal dementia (FTD), who showed an improvement in frontal lobe functions and a decrease in behavioral disturbance, thus slowing down cognitive decline [23].

Nevertheless, this study has some limitations, the main of which is the relatively low number of patients enrolled. Also, the study participants were recruited from a single hospital, so selection bias may exist. The promising results obtained show that larger-scale multicentric studies, with a double-blind design and a longer follow-up, are needed to confirm the usefulness of PEALut in the dietary supplementation of patients with acute ischemic stroke.

## 5. Conclusions

This prospective, randomized, controlled clinical trial confirmed the utility of dietary supplementation with PEALut in the management of patients with acute ischemic stroke. Starting PEALut supplementation from the first hours after the ischemic event allowed an improvement in disability along with a greater recovery of cognitive functions compared to thrombolysis alone, highlighting the importance of early treatment and opening the way to possible future uses in the management of other disabling conditions.

## Figures and Tables

**Table 1:** Patients baseline characteristics.

Baseline Characteristics	Control (N = 30)	PEALut (N = 30)	*p*
Days in Stroke Unit	10.2 ± 1.29	9.0 ± 1.27	0.3423 ^a^
NIHSS	10.8 ± 0.88	8.4 ± 0.61	0.5464 ^b^
BI	21.3 ± 3.06	27.8 ± 2.96	1.0000 ^b^
mRS	4.4 ± 0.12	3.9 ± 0.14	0.6819 ^b^
AIS site, n (%)			0.7413 ^c^
Posterior circulation	2 (7)	3 (10)	
Anterior circulation	28 (93)	27 (90)	
Previous stroke (without sequelae), n (%)	4 (13)	3 (10)	1.0000 ^c^
Previous TIA, n (%)	7 (23)	7 (23)	1.0000 ^c^
Thrombophilia, n (%)	1 (3)	0 (0)	1.0000 ^c^
Heart failure, n (%)	2 (7)	5 (17)	0.4238 ^c^
Ongoing neoplastic disease, n (%)	6 (20)	4 (13)	0.7306 ^c^
Ischemic heart disease, n (%)	5 (17)	7 (23)	0.7480 ^c^
Hypertension, n (%)	21 (70)	26 (87)	0.2092 ^c^
Non-ischemic heart disease, n (%)			0.0382 ^c^
Hypertensive	11 (37)	20 (67)	
Valve	0 (0)	0 (0)	
Dilative	0 (0)	0 (0)	
Other	4 (13)	4 (13)	
Presence of prosthesis valve COPD, n (%)	3 (10)	2 (7)	1.0000 ^c^
Epilepsy, n (%)	2 (7)	1 (3)	1.0000 ^c^
Current infections, n (%)	1 (3)	1 (3)	1.0000 ^c^
Chronic kidney disease, n (%)	0 (0)	0 (0)	1.0000 ^c^
Diabetes, n (%)			0.6130 ^c^
Insulin dependence	2 (7)	4 (13)	
NIDDM	5 (17)	3 (10)	

N, number of patients considered. AIS, Acute Ischemic Stroke; TIA, Transient Ischemic Attack; COPD, Chronic Obstructive Pulmonary Disease; NIDDM, Non-Insulin-Dependent Diabetes Mellitus; NIHSS, National Institutes of Health Stroke Scale; BI, Barthel Index; mRS, modified Rankin Scale; ^a^ Kruskal–Wallis test; ^c^ Fisher test; ^b^ post hoc analysis (Tukey–Kramer multiple-comparisons test). Values are presented as mean ± S.E.

**Table 2:** NIHSS score.

NIHSS Score	T0	T1	T2	*p*
Control	10.8 ± 0.88	6.3 ± 0.87	4.7 ± 0.74	0.7134
PEALut	8.4 ± 0.61	3.9 ± 0.64	2.3 ± 0.41	

Data are presented as mean ± S.E.

**Table 3:** BI score.

BI Score	T0	T1	T2	*p*
Control	21.3 ± 3.06	50.2 ± 4.72	64.6 ± 4.81	0.0020
PEALut	27.8 ± 2.96	64.2 ± 3.62	86.0 ± 3.06	

Data are presented as mean ± S.E.

**Table 4:** mRS score.

mRS Score	T0	T1	T2	*p*
Control	4.4 ± 0.12	3.3 ± 0.24	2.9 ± 0.29	0.0001
PEALut	3.9 ± 0.14	3.0 ± 0.20	1.3 ± 0.23	

Data are presented as mean ± S.E.

**Table 5:** Percentage of patients able to perform cognitive test in the two groups.

	Control		PEALut	
	**T1** (N = 30)	**T2** (N = 26)	**T1** (N = 29)	**T2** (N = 28)
MMSE	30%	19.2%	69%	75%
MoCA	26.7%	11.5%	65.5%	64.3%

MMSE, Mini-Mental State Examination; MoCA, Montreal Cognitive Assessment.

## Data Availability

Data are available under request to the corresponding author.
